# Molecular Biology of Ornamental Plants

**DOI:** 10.3390/plants12193493

**Published:** 2023-10-07

**Authors:** Aiping Song, Yu Chen

**Affiliations:** 1 State Key Laboratory of Crop Genetics & Germplasm Enhancement and Utilization, Key Laboratory of Landscaping, Key Laboratory of Flower Biology and Germplasm Innovation (South), Ministry of Agriculture and Rural Affairs, Key Laboratory of Biology of Ornamental Plants in East China, National Forestry and Grassland Administration, College of Horticulture, Nanjing Agricultural University, Nanjing 210095, China; 2 Zhongshan Biological Breeding Laboratory, No.50 Zhongling Street, Nanjing 210014, China; 3 College of Agro-Grassland Science, Nanjing Agricultural University, Nanjing 210095, China; cyu801027@njau.edu.cn

## 1. Summary

Relative to model plants, ornamental plants have many special characteristics, such as their flower color and shape, and a floral fragrance. Furthermore, these traits are unique and complex to different plants. With the development of sequencing technology and omics tools, core regulatory networks and genes related to specific traits were discovered in a variety of ornamental plants. These research methods have enriched the exploration of the regulatory mechanisms of ornamental traits and provided elite candidate genes for directional breeding. Our Special Issue “Molecular Biology of Ornamental Plants” covers 13 ornamental plant species, including 7 woody flowers, 3 perennial flowers, 1 aquatic flower and 2 herbs. The research topics include flower color, abiotic stress, species diversity, plant development and research protocol innovation. The research methods integrate the latest methods of bioinformatics and molecular biology. The following is a brief introduction to the main progresses in this Special Issue.

## 2. Main Progresses in this Special Issue

### 2.1. Molecular Evolution and Species Diversity

Zhang et al. investigated the TPS gene family in eight sequenced Rosaceae species through classification, chromosomal location, orthologous relationships and duplication analysis. The results showed that the distribution of the TPS gene family among Rosaceae species revealed an assortment of family numbers and functions, and further observed that lineage-specific expansions of the TPSs accompanied by frequent domain loss were widely observed within different TPS clades [1]. Zhang et al. conducted the transcriptome sequencing of *Luculia yunnanensis*. Then, the MISA (Microsatellite) tool was used to identify SSR loci from all unigenes, and a total of 15,384 SSRs were identified. Furthermore, 17 of the synthesized EST-SSR primers can be used for subsequent population genetic diversity analyses and molecular marker-assisted breeding [2]. Yuan et al. compared the population structure and genetic diversity of a distylous herb, *Hedyotis chyrsotricha* (Rubiaceae), in two contrasting island systems of southeast China. RAD-Seq data emphasized that the matrix of water in the island system may facilitate the seed (fruit) dispersal of *H. chrysotricha*, thus maintaining population connectivity and providing ongoing resilience to the lasting effects of habitat fragmentation [3].

### 2.2. Gene Function Research

Fei et al. identified and cloned two genes from the NCED family in *Paeonia lactiflora*, which are related to ABA synthesis in the herbaceous peony, named *PlNCED1* and *PlNCED2*. Further studies confirmed the function of the *PlNCED* gene in inducing seed dormancy and hindering seed germination [4]. In iris plants, Wang et al. confirmed the function of the AP2/ERF superfamily member IlAP2, which interacts with IlMT2a, in enhancing Cd tolerance [5]. Zhu et al. used transcriptome approaches to study the molecular mechanisms affecting the petal coloration of four varieties of *Rhododendron pulchrum*. The results showed that there were differentially expressed genes among the four different petal samples, including signal transduction-related genes, anthocyanin biosynthesis genes and transcription factors. The development of a red color may be related to a higher expression of *DFR* genes, which promotes the accumulation of anthocyanin [6]. Zhang et al. were involved in regulating plant branching and development to provide candidate genes for improving crop architecture through gene editing or directed breeding [7].

### 2.3. Gene Family Identification

Yang et al. identified 38 *OfXTH* genes belonging to the four main phylogenetic groups in *Osmanthus fragrans*. The gene structure, chromosomal location, synteny relationship and cis-acting elements and expression patterns were analyzed on a genome-wide scale. The expression patterns showed that most *OfXTHs* were closely associated with the flower-opening period of *O. fragrans*, and five of them were involved in the regulation of flower opening by responding to ambient temperature changes [8]. Cao et al. identified twenty-nine *NnDofs* in lotuses, of which the physicochemical properties vary; however, all of which contain conserved zinc finger structures. A promoter analysis, RNA-seq atlas and qRT-PCR were performed to demonstrate the potential function of *NnDofs* in the salt tolerance of lotuses [9]. Gu et al. identified 21 *LiNAC* genes from the transcriptome data in *Lagerstroemia indica*, and the physicochemical characteristics of amino acids, their subcellular localization, transmembrane structure, GO and KEGG enrichment, and expression patterns were examined. A further qRT-PCR indicated that these *LiNAC* genes may be involved in the regulation of weeping traits in *L. indica* [10]. Ma et al. identified 16 *MsHSF* genes in *Medicago sativa*, and the physicochemical properties, subcellular localization, synteny analysis, GO annotation and enrichment, and the protein interaction networks of amino acids were also examined. Similarly, the RNA-Seq results found that the *MsHSF* gene family plays a key role in abiotic stress [11]. Guo et al. performed a phylogenetic analysis of 120 AP2/ERF genes in the *Rhododendron simsii* genome; cis-acting elements involving plant growth regulators, the response to abiotic stress and MYB binding sites were detected in the upstream sequences of *RsAP2* genes. In addition, twenty *RsAP2* genes were selected for a qRT-PCR, and the results showed that most of the *RsAP2* genes responded to these abiotic stresses [12]. Liu et al. analyzed 204 *HhMYB* family members in *Hibiscus hamabo* using RNA-seq and a qRT-PCR, and found that *HhMYB* was involved in plant responses to salt and drought stress to varying degrees [13]. Song et al. identified and analyzed 25 *SWEET* genes in *Rosa chinensis* ‘Old Blush’, and through a transcriptome analysis, *SWEET2a* and *SWEET10c* were found to be candidate genes involved in the rose’s ability to tolerate cold conditions [14].

### 2.4. Research Protocol Innovation

Continuous updates in technology and tools promote further studies of ornamental plants. Liu et al. selected 14 candidate genes from the transcriptome database of *Lindera megaphylla* for additional qRT-PCRs under different conditions to further verify the reliability of selected reference genes above [15]. Cheng et al. developed an efficient virus-induced gene silencing (VIGS) system using the leaf tip needle injection method, which could effectively silence genes in *Lycoris chinensis*, providing a powerful tool for gene function studies [16].
